# Cases of Fever

**Published:** 1826-10

**Authors:** 

**Affiliations:** St. George's Hospital


### FEVER.

### *Cases of Fever, treated at* St. George's Hospital, *by* Dr. Chambers; *with a few Practical Remarks.*

The advantage derived from the free use of calomel, assisted by more direct purgatives, in the treatment of the common continued fever of this country, is now almost universally acknowledged. In fact, those who have been in the habit of treating this disease, must have observed that, in most instances, when purgatives have been early and steadily administered, all the symptoms have in a short time yielded to them.

The following case is an example of the effect produced by this simple treatment.

George Pringley, ætatis thirty, coachman, was admitted on the 23d of August. He complained of a sensation of weight in his head, with unusual dullness of intellect, and of nausea and want of appetite; he appeared much emaciated. His pulse was 120, small; his skin dry and hot; his tongue was covered with a thick fur in the centre, its edges being of a bright red colour; his bowels were irritable; his stools dark, slimy, and offensive; and his abdomen full and hard.

He said he had been ill eleven days with the above-mentioned symptoms, which followed an attack of shivering.

> Twelve leeches were applied immediately to his forehead and temples. He was placed on "fever diet,"* and was ordered to take the following medicines:—℞. Hydrarg. Submur. gr. v.; Pulv. Antimon. gr. iv. M. fiat pulvis omni nocte sumendus.—℞. Aquæ Pimentæ ℥jss.; Rhei Pulveris ℈j. M. fiat haustus omni manè sumendus.—Sumat Haustum Salinum sextâ quâque horâ.

Two days afterwards he was much better, and was then ordered to take his calomel and James's powder every other night, and the rhubarb draught the following mornings.

On the 28th, (three days more,) he was free from all uneasiness in his head; his tongue was clean and moist; his skin was cool; his motions healthy; and his appetite improved. He now complained only of debility, to which the frequency of his pulse (still beating about 100 in a minute,) was attributable. He took the following draught for a few days: viz.

> ℞. Infusi Cascarillæ ℥xss.; Acidi Nitrici diluti ʒss.; Syrupi ℥j. M. fiat haustus ter die sumendus.

His diet was gradually improved until he was discharged cured, on the 3d of September.

* This diet consists chiefly of farinaceous decoctions.

It would be easy to multiply instances of the efficacy of this mode of treatment in a great majority of cases of fever, even when the remedies in question have not been prescribed until the second or third week of the disease.

In spite, however, of the great advantage derived from this practice in general, it is well known that sometimes a case of fever will occur, in which, although most of the symptoms may be more or less mitigated by the free use of these evacuants, yet, after all, it will be obvious that the patient is still suffering from the irritation of fever. It is under these circumstances that mercury, exhibited in small and repeated doses, appears to exert the most decided influence over the disease. It is, therefore, very important to ascertain, if possible, what are precisely the symptoms in the progress of continued fever, which should induce the practitioner to change his mode of treatment, by abandoning the use of direct purgatives, and adopting the alterative practice just mentioned.

The subjoined cases may, perhaps, contribute towards the elucidation of this point. In the first of these it will be seen that the original febrile action was much mitigated by the early treatment, but that there still remained unremoved some source of constitutional disturbance, which afterwards yielded to the alterative influence of mercury.

Thomas Kelly, aged forty-five, labourer, was admitted into the hospital on the 9th of August, 1826. He complained of headache and confusion of intellect, and of fulness and heavy pain of the abdomen, particularly about the epigastrium and hypochondria; the pain was increased on firm pressure being made over the parts affected. His pulse was 110, and full; his skin hot, his tongue furred; and his bowels open. The disease was of ten days' standing.

He was ordered to have fever diet; to lose twelve ounces of blood immediately from the arm; and to take the following medicines: viz.—Calom. gr. v. every night, and a Senna Draught every morning.

The blood drawn was not inflamed, but the crassamentum was large in proportion to the serum, and very firm.

He continued the purgatives until the 14th instant, on which day he had no pain or uneasiness either in the head or abdomen: his pulse was eighty, soft; his skin cool, his tongue moist, and his bowels open. His only complaint was debility.

The Calomel and purgative medicines were now left off, and he was ordered to take thrice daily a draught consisting of Mistura Camphoræ, with a few drops of Spiritus Ammoniæ Aromaticus.

The next day, however, although he had no return of pain or uneasiness, and no acceleration of the pulse, yet his skin was again very hot, his tongue had become dry and somewhat furred, and his general aspect was dull and heavy.

He was now ordered Hydrarg. Submur. gr. iij.; Pulv. Antim. gr. iij. M. fiat pulv. quartâ quâque horâ sumendus.—Repetatur Mist. Camphoræ c. Spiritû Ammoniæ Aromatico.—Applicetur Emplastrum Cantharidis nuchæ.

He continued the powder, gradually diminishing the daily quantity of the calomel as the symptoms improved, until the 25th. He had then recovered his natural manner, and every function was restored to its healthy condition. His mouth was not affected by the mercury.

After taking for a few days the following tonic draught —

℞. Infusi Cascarillæ ℥jss.; Ammoniæ Carbonatis gr. v. M. fiat haustus ter quotidie sumendus, he was discharged cured.

It is not intended here to enter at length into the pathology of these changes. It will suffice to say that, when this cessation, or distinct mitigation, of the early symptoms of the disease takes place, it is probable that the primary fever has been subdued, and that what remains of constitutional disturbance depends on those organic lesions which are now well ascertained to be ordinary consequences of continued fever. These lesions, in their turn, excite a new febrile action in the system, which ought by no means to be considered as a relapse of the original disease. It is, in fact, a secondary fever, depending on the irritation produced by structural injury, and is clearly distinguishable from the primary disease by its remittent or hectic character, as well as by the extraordinary irritability, joined with prostration of strength, which accompanies it after it is established.

In the case which has been just related, it may be presumed, from the readiness with which the symptoms yielded to the treatment, that the secondary disease was in its infancy; and that the injury of structure was as yet very limited in its extent.

It happens, however, occasionally, that, long before the primary disease has been overcome, the secondary irritation has commenced; so that, when the former is afterwards subdued, the latter presents itself to us in an aggravated form, and is besides the more intractable, because the strength of the patient is proportionally diminished.

The following case is an example of this variety of the disease. It will be seen that, in this instance, whilst the primary fever was as yet unsubdued, the constitution was suffering much additional irritation from the injury early inflicted on the mucous membrane of the bowels; so that, when the symptoms of the original disease were at length relieved by the treatment, an irritative fever of considerable intensity remained, which required great caution and delicacy in its treatment, and only yielded, after many days, to the alterative effect of mercury.

Maria Green, aged thirty, a single woman, living in Pimlico, was admitted into St. George's Hospital, on the 7th of August, with the symptoms of severe continued fever; amongst which were most remarkable the pain and confusion of the head, the great frequency of the pulse, the unusual fulness and hardness of the abdomen, and the frequent passage of dark, slimy, and offensive motions. These symptoms, she said, had commenced eight days before her admission.

She was placed on fever diet, and treated, for the first few days after she came into the hospital, with Calomel, Senna, and Antimonial Wine in Saline Draughts; and leeches were applied to her abdomen, and also to her forehead and temples.

In a few days these remedies relieved the pain in the head, removed the fulness and hardness of the abdomen, and rendered the motions of a better colour. But, nevertheless, there remained considerable anxiety of the countenance, and some unsteadiness of the mind, with extreme debility and deafness. The pulse was still very frequent (130), and occasionally *beat doubly*. The skin, although sometimes cool, was at irregular intervals (generally twice a-day) pungently hot, and the cheeks were then brightly flushed; after which she was covered with a profuse hot perspiration. The tongue gradually lost its fur, but became of a bright red colour, with a smooth, glazed, and viscid surface, which after a time was spotted with aphthous vesicles. Although the fulness of the abdomen had subsided, yet there was a degree of tension still remaining; and sharp pain was felt towards the right iliac region when firm pressure was made over that part. The motions were frequently liquid, of a bright yellow colour, and a very offensive smell. Added to this, there was at intervals a distressing irritability of the stomach, with vomiting of green bilious fluid.

When this train of symptoms, which clearly showed that the ulcerative process was going on in the ileum and cœcum,* presented themselves, the patient was ordered to take the following pill:

℞. Hydr. c. Cretâ gr. ij.; Extr. Papav. albi gr. iij. M. fiat pilula sextâ quâque horâ sumenda.

The irritability of the bowels was from time to time so far restrained by small quantities of opium, and by astringent enemata, as to prevent excessive exhaustion, but nevertheless several mo-

* It may be worth while to remark, that the train of symptoms mentioned as characterising ulceration of the mucous lining of the bowels, would have been complete without the tenderness on pressure which was present in this case; for extensive destruction of the interior of the bowels, about the ileo-cœcal valve, is often found after death, although there has been previously no tenderness on pressure. In fact, this symptom is not present, except when the ulceration of the mucous membrane has excited some irritation in the neighbouring peritoneum.

tions were passed daily; and a small quantity of port wine, with water, was now and then administered under circumstances of extraordinary exhaustion. An opiate plaster was also applied to the abdomen. These latter expedients, however, were used merely as palliatives; but the mercurial pill was considered to constitute the essential part of the treatment, and was therefore steadily continued from the 13th to the 27th of August.

On the 27th, the gums were affected by the mercury.* The irritability of the bowels now at once entirely subsided: she had on that day only one motion, and that of a natural character. The pulse, which was just before 120, was now only ninety; the skin was cool throughout the day; the tongue became moist, and of a healthy surface and colour; the countenance was composed; the mind also was clear and tranquil, and the appetite returned.

She then left off the Mercurial Pill, and took only a small quantity of Castor-oil occasionally; and thrice a-day a Saline Draught, with three minims of Tincture of Opium.

Since that time she has been almost uniformly improving; can now eat animal food with appetite; and will probably leave the hospital in a short time.

It will be observed, that in the last case it is taken for granted that the patient has survived ulceration of the mucous coat of the bowels. That this is not a very uncommon occurrence, is now well established by the dissection of those who have accidentally died some time after they have been convalescent from fever, and in whom the cicatrization of ulcers in the bowels has been very clearly seen. It is not, however, intended here to enter into this question, respecting which, indeed, those who are acquainted with the subject will have no doubt or difficulty.

* Soreness of the gums is by no means indispensable; for the good effects of this treatment are often fully exhibited, in the relief of the symptoms, without any tenderness of the mouth.

